# London County Council and Tuberculosis

**Published:** 1913-11-08

**Authors:** 


### LONDON COUNTY COUNCIL AND TUBERCULOSIS.

### Position of the London Hospitals.

Some interesting statements as to the attitude of the London hospitals towards the London County Council's scheme for the treatment of tuberculosis in London were made at Tuesday's meeting of the County Council. The Public Health Committee's scheme proposes that the dispensary treatment of uninsured persons shall be undertaken by the Borough Councils, the County Council retaining a certain measure of control by making a contribution of 25 per cent. of the total cost. It transpired during the debate that a deputation representing the London General Hospitals has waited upon the Public Health Committee, urging that, while they were anxious to fall in with the scheme, the County Council ought to be the one central authority. There was, however, a general feeling that to avoid any further delay in preparing a scheme, the Borough Councils should be allowed to arrange for the provision of dispensary treatment in their own areas.

Mr. A. L. Leon said that twelve big hospitals had come before the committee, and were anxious to fall in with the scheme, but they insisted that the County Council should be the one central authority for London. They had had cases in which borough councils had refused to act with hospitals, and the hospitals had refused to act with borough councils. Such difficulties could be overcome by having one central authority.

Mr. Frank Briant declared that no more serious blow would ever be struck than if the hospitals were left out of any scheme to deal with tuberculosis. Hospitals with medical schools—in fact, the whole of the general hospitals—had definitely sent them a resolution, in which they emphasised the importance of the administration of the scheme being put into the hands of the London County Council. If the borough councils decided to have nothing to do with the hospitals, what would happen? They would have a tuberculosis officer at, say, £500 a year—not a man of an enormous practice at that rate of salary. What would happen in cases where tubercular disease was accompanied by other forms of disease? Would the tuberculosis officer treat them entirely, or were they to be treated partly at the hospitals? For the past eight and a half months St. Thomas's Hospital had had a most excellent dispensary treatment. They had treated 730 people, and out of those 730 no less than 340 had to have special treatment of one kind or another—surgical, dental, throat, or *x*-rays—which was open to them at a hospital, but not at a dispensary of the ordinary kind. If the borough councils refused to do what they wanted and what the hospitals wanted, the whole cause of tuberculosis treatment would suffer for many years to come. The Council should pluck up courage and take the whole responsibility into its own hands, for they had a great opportunity but were losing it.

Mr. J. A. Dawes, M.P., Chairman of the London Insurance Committee, observed that they had a considerable grievance against the Local Government Board, but rather than have any more delay they had much better pass the scheme, and give the borough councils the chance of formulating schemes in their own districts. He had not gathered that the representatives of the leading hospitals made it an essential condition that the County Council should be the one authority. He gathered that they would be willing to try and make the best of a bad scheme, if the borough councils would work with them. He did not like the scheme, but it was the best they could do.

Mr. O. E. Warburg said that under the scheme the borough councils and the hospitals would have every opportunity of co-operation, and the function of the Council would be to see that a proper balance was maintained between dispensaries, hospitals, and the local medical officers of health.

After considerable discussion the Public Health Committee's scheme, as far as the dispensary treatment of uninsured persons was concerned, was agreed to.

With regard to the provision of residential accommodation for uninsured persons, the Council decided that arrangements would be made, if possible, with the Metropolitan Asylums Board, with hospitals, and with sanatoria for the provision of the accommodation required. It was referred to the Public Health Committee to report whether adequate provision could be made in this manner without the Council being called upon itself to own and manage institutions.

Mr. Dawes said that the Asylums Board had done all they could to help the Insurance Committee out of a serious difficulty, but the accommodation at their disposal was not really suitable.

